# Proteomic profiling identifies an oncogene ITGA2 and its downstream targets in gastric cancer

**DOI:** 10.1007/s12094-026-04231-w

**Published:** 2026-02-02

**Authors:** Michelle Xin Liu, Kent-Man Chu

**Affiliations:** 1 Department of Surgery, The University of Hong Kong, Pokfulam, Hong Kong, H.K. China; 2 Department of Surgery, Queen Mary Hospital, Pokfulam, L9-57, Laboratory Block, 21 Sassoon Road, Hong Kong, H.K. China

**Keywords:** Exosomes, Gastric cancer, ITGA2, Oncogene

## Abstract

**Purpose:**

Gastric cancer is a leading cause of cancer-related mortality worldwide, with metastasis being the primary cause of death. Exosomes secreted by tumor cells are key mediators of intercellular communication and can prepare distant sites for metastasis by altering the local microenvironment. We hypothesized that exosomes released by gastric cancer cells deliver cargo that regulates specific proteins in recipient gastric cancer cells, thereby enhancing tumor progression and metastatic potential.

**Methods:**

Human gastric cancer cell lines were treated with exosomes isolated from the conditioned medium of other gastric cancer cells. Differential proteomic analysis was performed to identify proteins significantly altered by exosome treatment. ITGA2 expression was validated in exosome-treated cells and in clinical gastric cancer tissues versus adjacent normal tissues using RT-qPCR and western blotting. The functional role of ITGA2 was assessed by siRNA-mediated knockdown, followed by MTT proliferation assays and fluorometric transwell assays for migration and invasion. A second proteomic analysis was conducted on ITGA2-knockdown versus control cells to identify downstream targets and affected pathways.

**Results:**

Exosome treatment significantly upregulated ITGA2 expression in recipient gastric cancer cells. ITGA2 was also markedly overexpressed in human gastric cancer tissues compared with adjacent normal mucosa. Knockdown of ITGA2 significantly suppressed cell proliferation, migration, and invasion. Proteomic profiling of ITGA2-knockdown cells revealed numerous differentially expressed proteins enriched in pathways related to cell adhesion, motility, extracellular matrix organization, and intercellular signaling.

**Conclusion:**

Exosomes derived from gastric cancer cells induce ITGA2 overexpression in recipient tumor cells, where ITGA2 functions as an oncogene that promotes proliferation, migration, and invasion. The downstream targets of ITGA2 implicate multiple pro-tumorigenic signaling networks, suggesting that the exosomes–ITGA2 axis may represent a novel therapeutic target in gastric cancer progression and metastasis.

**Supplementary Information:**

The online version contains supplementary material available at 10.1007/s12094-026-04231-w.

## Introduction

Gastric cancer (GC) ranks as the fifth most commonly diagnosed malignancy and the fifth leading cause of cancer-related mortality worldwide, with approximately 970,000 new cases and 660,000 deaths reported in 2022 [1]. It shows substantial geographical variation in incidence, with higher rates in Asia, South America, and Eastern Europe [2, 3], where age-standardized incidence rates remain among the highest globally. In Hong Kong, GC was the sixth leading cause of cancer death in 2023, accounting for 594 fatalities [4]. Radical gastrectomy combined with perioperative chemotherapy represents the cornerstone of curative-intent treatment for localized disease. Nevertheless, a substantial proportion of patients develop recurrence or distant metastasis even after potential curative resection, and therapeutic options for metastatic GC remain limited, resulting in poor prognosis of patients with metastasis. Metastasis is therefore the principal determinant of poor prognosis in GC.

Exosomes, small extracellular vesicles (30–150 nm) with lipid bilayer membranes, are stable in biological fluids and serve as critical mediators of intercellular communication [5]. Tumor-derived exosomes transfer proteins, lipids, and nucleic acids to recipient cells, thereby reprogramming the local and distant microenvironment to facilitate pre-metastatic niche formation, immune evasion, angiogenesis, and metastatic outgrowth [6, 7]. In GC, multiple studies have demonstrated that tumor-derived exosomes promote progression and metastasis through diverse mechanisms [8], including activation of NF-κB in macrophages [9] and upregulation of adhesion molecules in peritoneal mesothelial cells [10]. In addition, LC–MS/MS (liquid chromatography–tandem mass spectrometry) had been applied to detect the proteomic profile of exosomes from the serum of patients with gastric cancer. For example, a study reported that tripartite motif-containing protein 3 (TRIM3) in exosomes of serum of gastric cancer patients was lower than in healthy controls [11]. The higher expression of exosomal transforming growth factor beta 1 (TGF-β1) in gastric cancer patients has been analyzed to be associated with TNM stage and lymph node metastasis, correlated with forkhead box protein 3 + (FOXP3 +) Treg cells [12]. Furthermore, exosomal angiotensinogen (AGT), serpin family H member 1 (SERPINH1), and matrix metallopeptidase 7 (MMP7) have been demonstrated to perform well in predicting overall survival and be non-invasive prognostic biomarkers of gastric cancer [13]. Gastrokine 1 (GKN1) has also been identified to be secreted from HFE-145 gastric epithelial cells and can reduce tumor growth and tumor volume, which could be served as a therapeutic target for gastric cancer [14], while these effective therapeutic proteins can be encapsulated into the exosomes and might prevent gastric cancer progression [15]. Thus, these findings collectively underscore the pivotal role of exosomal cargo in GC pathogenesis.

Our group previously established protocols for isolating exosomes from both GC patient serum and cell culture medium, confirming their identity by western blotting, transmission electron microscopy, and nanoparticle tracking analysis [16]. We further showed that exosomal miRNAs are functionally transferred to recipient cells [17, 18]. Building on this foundation, the present study employed homologous exosome treatment (AGS cells treated with BCG23-derived exosomes and vice versa) followed by label-free quantitative proteomics to identify proteins consistently altered across both cell lines. Integrin alpha-2 (ITGA2) emerged as one of the most strongly and reproducibly upregulated candidates.

ITGA2 encodes the α2 subunit of the α2β1 integrin, a major collagen/laminin receptor that mediates cell–extracellular matrix adhesion, migration, and signaling. ITGA2 is overexpressed and functions as an oncogene in multiple malignancies, including breast, colorectal, hepatocellular, lung, ovarian, and pancreatic cancers [19–25], where it promotes epithelial–mesenchymal transition, invasion, and therapeutic resistance. Although The Human Protein Atlas and other public datasets indicate elevated ITGA2 expression in GC tissues relative to normal gastric mucosa, the regulation of ITGA2 by tumor-derived exosomes and its functional contribution to gastric carcinogenesis have not been previously investigated.

Here, we reported that GC-derived exosomes induced ITGA2 overexpression in recipient tumor cells, where ITGA2 acted as a potent driver of proliferation, migration, and invasion. We further delineated the downstream proteomic alterations and signaling pathways governed by ITGA2. To our knowledge, this is the first study to establish the exosomes–ITGA2 axis in gastric cancer, offering new mechanistic insights into metastatic progression and identifying ITGA2 as a potential therapeutic target.

## Materials and methods

### Human tissue samples

40 pairs of human gastric cancer and non-tumor tissue samples were collected directly after the surgical resection at Queen Mary Hospital, Hong Kong. All of the tumor tissue samples were validated to be malignant by experienced pathologist. All of the samples were obtained with the participants’ informed consent and none of the patients received preoperative treatment. All samples were immediately frozen in liquid nitrogen and stored at − 70 °C.

### Cell lines and cell culture

Human gastric cancer cell lines AGS and NCI-N87 (ATCC, Rockville, MD, USA), MKN28, MKN45, and MKN7 (RIKEN, Japan), and BCG23 and PAM82 (from Beijing Cancer Institute) were used in this study. Cell lines AGS and BCG23 were established from gastric cancer tissues of original site (stomach). MKN7 was established from gastric cancer metastasized to lymph node. MKN28, MKN45, and NCI-N87 were established from gastric cancer metastasized to liver. Cells were cultivated in RPMI1640 medium (Gibco BRL, Gaithersburg, MD, USA) supplemented with 10% exosome-depleted fetal bovine serum (FBS) (SBI, System Biosciences, USA). All cells were incubated at 37 °C in a humidified incubator which contains 5% CO_2_ [26].

### Cell treatment with culture medium containing exosomes

Two gastric cancer cell lines AGS and BCG23 were used in this treatment. Both cell lines were established from gastric cancer of the original site (stomach, not from the metastatic sites). Both cell lines were cultured in RPMI1640 with exosome-depleted FBS. 10 ml of culture medium for culturing AGS or BCG23 cells were collected 6 days after cell seeding. Exosomes in the culture medium were extracted using ExoQuick-TC from SBI (System Biosciences, USA). The exosomes from AGS cell culture medium were used to co-incubated with BCG23 cells. While the exosomes from BCG23 cell culture medium were used to co-incubated with AGS cells. Proteins in AGS or BCG23 cells were extracted after 5–7 days of incubation of exosomes. The proteins were then applied for proteomic profiling.

### Reverse transcription-quantitative polymerase chain reaction

Total RNAs were reverse-transcribed to cDNA using Omniscript RT Kit (QIAGEN, USA) according to the manufacturer’s instructions. Real-time PCR was performed using miScript SYBR Green PCR Kit (QIAGEN). Specific primers (ITGA2 F:5’-TTGCGTGTGGACATCAGTCTGG-3’; R:5’-GCTGGTATTTGTCGGACATCTAG -3’; β-actin F: 5’-CACCATTGGCAATGAGCGGTTC-3’; R: 5’-AGGTCTTTGCGGATGTCCACGT-3’) for q-PCR were provided by IDT (Singapore). Q-PCR was performed in ABI ViiA7 Real-time PCR system (Applied Biosystems, Waltham, MA, USA). The expression of mRNAs was normalized to β-actin using the 2-ΔΔCt method. ΔCt was calculated by subtracting the Ct values of β-actin from the Ct values of targets. ΔΔCt was then calculated by subtracting ΔCt of non-tumor sample from ΔCt of the paired tumor sample. Fold changes in the gene were calculated by the Eq. 2 − ΔΔCt.

### Transfection of siRNA of ITGA2 in gastric cancer cells

The specific siRNA of ITGA2 (ON-TARGETplus Human ITGA2 siRNA, SMARTPool, 5 nmol, L-004566-00-0005) was purchased from Dharmacon™ (California, USA). 1X10^5^ cells were seeded into a 6-well plate a day in advance of transfection and transfected with 20 nM siRNA or scrambled control using Hiperfect Transfection Reagent (QIAGEN, Hilden, Germany), following the manufacturer’s instructions. Transient transfected cells were applied for evaluation of expression levels and functional assays.

### Cell proliferation assay

MTT assay was used for cell proliferation assay. 5000 cells/well were seeded in 96-well plates with incubation of ITGA2-enriched exosomes or control exosomes. After 24 and 48 h, the culture medium was discarded and re-stained with 3-(4,5-dimethyl-2-thiazolyl)-2,5-diphenyl-2H-tetrazolium bromide (MTT) (5 mg/ml) for 3 h. Absorbance was measured at 570 nm on a microplate reader.

### Cell migration and invasion assay

Cells with siRNA of ITGA2 or control for 24 h were harvested and suspended in RPMI 1640 medium. A transwell culture insert with 8 μm pore size membrane for 24-well plate (Cat no. CBA-101-C, Cell Biolabs Inc., CA, USA) was used to analyze the migration activity. 500 μl of RPMI containing 10% FBS was added to the lower well, and 2 × 10^5^ suspension of cells in 300 μl of serum-free RPMI 1640 medium were added into each insert. The plate was incubated at 37 °C for 24 h. After incubation, the media of the inserts were carefully aspired. Then the inserts were transferred to clean wells containing 225 μl of Cell Detachment Solution and incubated for 30 min at 37 °C. After that, 75 μl of 4X Lysis Buffer/CyQuant® GR dye solution was added to each well containing cells and 225 μl of Cell Detachment Solution. The plate was incubated for 20 min at a room temperature. Finally, 200 μl of the mixture in each well was transferred into a 96-well plate and red with a fluorescence plate reader at 480 nm.

Invasion assay was performed similarly by the method of migration assay described above except that matrigel-coated transwell chambers with 8 μm pore size membrane for 24-well plate (Cat no. CBA-101-C, Cell Biolabs Inc., CA, USA) was applied and the incubation time was 48 h.

### Western blot

Western blot was performed to evaluate the protein expression of ITGA2 in gastric cancer cells and tissue samples. Briefly, proteins were extracted and lysed by RIPA Buffer (Sigma Chemical Co., St Louis, MD, USA). Samples containing equal amounts of protein were separated by SDS-PAGE and electro blotted onto Immobilon-P Transfer Membrane (Applied Biosystems). The membrane was blocked with 5% no-fat milk, followed by incubation with antibodies specific for anti-ITGA2 (1:1000) and anti-β-actin (1:10,000, Cell Signaling Technology, Beverly, MA, USA), respectively. Blots were then incubated with anti-rabbit or anti-mouse secondary antibody conjugated to horseradish peroxidase (Amersham Pharmacia, Cleveland, OH) accordingly. The signals were captured by FUJI Medical X-Ray Film (Fuji, Japan) and developed by the FUJI system.

### Label-free quantitative proteomics (DIA)

The EasyPep Mini MS Sample Prep Kit was purchased from Thermo Fisher. Formic acid was purchased from Fisher Scientific. All solvents were in LC–MS grade equivalent or higher. Proteins were extracted in the EasyPep lysis buffer (100 μl per min. 1 million cells) with universal nuclease. Protein lysate was then quantified using BCA assay (Thermo Pierce), whereby 100 μg from each sample was used for reduction and alkylation at 95 °C for 10 min. After cooling to room temperature, the proteins were subjected to LysC / trypsin digestion at 37 °C for 180 min. The digestion was stopped by acidification, and the peptides were washed and desalted using the spin column provided in the kit, before they were analyzed by LC–MS/MS analysis.

Eluted peptides (200 ng per injection) were analyzed with nanoelute. UHPLC was coupled to Bruker timsTOF pro mass spectrometer. The peptide mixture was loaded onto an Aurora C18 UHPLC column (IonOpticks, Australia). Chromatographic separation was carried out using a linear gradient of 2–37% of buffer B (0.1% FA in ACN) at a flow rate of 300 nl/min over 100 min. MS data were collected over a m/z range of 100 to 1700, and MS/MS range of 100 to 1700. During MS/MS data collection, each TIMS cycle was 1.1 s and included 1 MS + an average of 10 PASEF MS/MS scans. To perform DIA, the instrument control software (Bruker timsControl v3.0) was extended to define quadrupole isolation windows as a function of the TIMS scan time (diaPASEF). The instrument control electronics were modified to allow seamless and synchronous ramping of all applied voltages. Eight windows for single 100 ms TIMS scans were defined up. Each of the four diaPASEF scans was done twice in the high-sensitivity scheme, and this resulted in one MS1 and eight diaPASEF scans per acquisition cycle. In both scan modes, the collision energy was ramped linearly as a function of the mobility from 59 eV at 1/K_0_ = 1.6 Vs cm^−2^ to 20 eV at 1/K_0_ = 0.6 Vs cm^−2^. To visualize the isolation of precursor ions, the collision energy was set to 5 eV to prevent fragmentation. Distributed 14 diaPASEF windows to one TIMS scan each and defined 14 × 50 Th precursor isolation windows from m/z 325 to 1,025. Also, defined 32 × 25 Th isolation windows from m/z 400 to 1,200. To adapt the MS1 cycle time in diaPASEF, the repetitions to 2 in the 16-scan diaPASEF scheme was set, and to 4 in the 4-scan diaPASEF scheme.

Raw mass spectrometry data were processed using Spectronaut. Direct DIA data analysis was performed on Spectronaut v.14 using default settings without N-acetyl variable modification enabled. Trypsin specificity was set to two missed cleavages and a protein and PSM false discovery rate of 1%; respectively. Data filtering was set to Q value and normalization set to global normalization.

### Statistics

Statistical analysis was carried out using Statistical Package for Social Sciences (SPSS) 26.0 for Windows (SPSS Inc., Chicago, IL, USA). Wilcoxon sign-rank test was applied for analysis of expression differences in paired tissue samples. Student’s t test was used to analyze the results expressed as mean ± SD. Differences were considered significant when *P* ≤ 0.05.

## Results

### Proteomic profiling screening in gastric cancer cell lines

1,000,000 treated gastric cancer cells were used for proteomic profiling. Proteomic profiling was performed with label-free proteomics and desalting, long gradient LC–MS/MS, using timsTOF Pro. Label-free quantitative proteomics (DIA) was applied for data analysis. Analysis of proteomic profiling revealed a list of 1001 differentially expressed proteins in AGS cells and 126 differentially expressed proteins in BCG23 cells. Further analysis revealed a list of 39 proteins overlapping in AGS and BCG23 cell lines from proteomic profiling (Table 1). ITGA2 was one of the overlapping proteins in both cell lines. It has been reported that ITGA2 functions as an oncogene in various cancers. It suggested that ITGA2 played an oncogenic role in primary gastric cancer and executes its oncogenic roles in recipient cells through exosomes. Therefore, ITGA2 was selected for further study.

**Table 1:** A list of 39 proteins overlapping in AGS and BCG23 cell lines from proteomic profiling

Protein IDs	Protein names	Gene names	Mol. weight [kDa]	*p* value
Q96QD8	Sodium-coupled neutral amino acid transporter 2	SLC38A2	56.025	0
P17301	Integrin alpha-2	ITGA2	129.29	0.0115
O95793	Double-stranded RNA-binding protein Staufen homolog 1	STAU1	63.182	0.015333333
Q9HBM0	Vezatin	VEZT	88.664	0.037377049
Q13415	Origin recognition complex subunit 1	ORC1	97.349	0.039752577
A3KN83	Protein strawberry notch homolog 1	SBNO1	154.31	0.040307692
Q16822;P35558	Phosphoenolpyruvate carboxykinase [GTP], mitochondrial	PCK2	70.698	0.0405
Q9UBU8	Mortality factor 4-like protein 1	MORF4L1	41.473	0.040622222
Q02818	Nucleobindin-1	NUCB1	53.879	0.040753623
P49591	Serine–tRNA ligase, cytoplasmic	SARS	58.777	0.040913043
Q14980	Nuclear mitotic apparatus protein 1	NUMA1	238.26	0.041098039
Q9Y6N5	Sulfide:quinone oxidoreductase, mitochondrial	SQRDL	49.96	0.041292308
Q99828	Calcium and integrin-binding protein 1	CIB1	21.703	0.041352941
Q96DX5	Ankyrin repeat and SOCS box protein 9	ASB9	31.857	0.041362637
Q15043	Zinc transporter ZIP14	SLC39A14	54.212	0.041591837
P41250	Glycine–tRNA ligase	GARS	83.165	0.041714286
Q9P0J1	[Pyruvate dehydrogenase [acetyl-transferring]]-phosphatase 1, mitochondrial	PDP1	61.053	0.042428571
Q15758	Neutral amino acid transporter B(0)	SLC1A5	56.598	0.042432432
Q8N5I9	Uncharacterized protein C12orf45	C12orf45	20.123	0.042526316
P09601	Heme oxygenase 1	HMOX1	32.818	0.042603175
Q53EL6	Programmed cell death protein 4	PDCD4	51.735	0.042666667
P54577	Tyrosine–tRNA ligase, cytoplasmic;Tyrosine–tRNA ligase, cytoplasmic, N-terminally processed	YARS	59.143	0.043013699
Q9UL33	Trafficking protein particle complex subunit 2-like protein	TRAPPC2L	16.145	0.043578947
P49588	Alanine–tRNA ligase, cytoplasmic	AARS	106.81	0.043666667
Q06609	DNA repair protein RAD51 homolog 1	RAD51	36.966	0.043801653
O95235	Kinesin-like protein KIF20A	KIF20A	100.28	0.044373832
Q9Y6M5	Zinc transporter 1	SLC30A1	55.299	0.044595745
Q96G74	OTU domain-containing protein 5	OTUD5	60.625	0.04519685
Q9NZN4	EH domain-containing protein 2	EHD2	61.161	0.045230769
Q96AZ6	Interferon-stimulated gene 20 kDa protein	ISG20	20.363	0.0455
Q8NFW8	*N*-Acylneuraminate cytidylyltransferase	CMAS	48.379	0.045555556
Q92597	Protein NDRG1	NDRG1	42.835	0.046198473
Q16555	Dihydropyrimidinase-related protein 2	DPYSL2	62.293	0.046352941
O00762	Ubiquitin-conjugating enzyme E2 C	UBE2C	19.652	0.046577778
Q14789	Golgin subfamily B member 1	GOLGB1	376.01	0.046666667
P32004	Neural cell adhesion molecule L1	L1CAM	140	0.04728125
Q15154	Pericentriolar material 1 protein	PCM1	228.54	0.047894737
Q06210	Glutamine–fructose-6-phosphate aminotransferase [isomerizing] 1	GFPT1	78.806	0.048744186
Q9Y4L1	Hypoxia up-regulated protein 1	HYOU1	111.33	0.049628571

### ITGA2 was significantly upregulated in gastric cancer cell lines and tissue samples

**Fig. 1:**
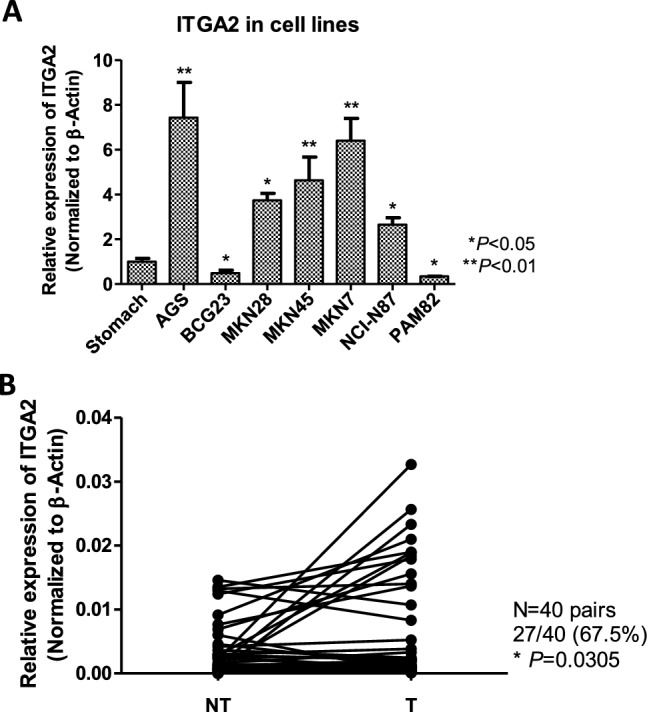
Expression of ITGA2 in gastric cancer cell lines and tissue samples. **A** Expression of ITGA2 was significantly upregulated in gastric cancer cell lines AGS, MKN28, MKN45, MKN7 and NCI-N87, comparing with a commercial normal stomach tissue sample (Fig. 1A, *P < 0.05, **P = 0.01). **B** Expression of ITGA2 was significantly upregulated in gastric cancer tissue samples (Fig. 1B, *N* = 40 pairs, NT: Non-Tumor tissue, T: Tumor Tissue; upregulated in 27 out of 40 pairs, 67.5%, *P = 0.0305)

Expression of ITGA2 was evaluated in gastric cancer cell lines and paired gastric cancer tissue samples by RT-qPCR. The results showed that ITGA2 was significantly upregulated in gastric cancer cell lines comparing with the expression of ITGA2 in gastric normal tissue sample (5 out of 7 cell lines, *P < 0.05, **P < 0.01, Fig. 1A). The expression of ITGA2 was also upregulated in gastric cancer tissue samples (27 out of 40 pairs, 67.5%, *P = 0.0305, Fig. 1B).

### Endogenous and exosomal expression of ITGA2 in cell lines and tissue samples

**Fig. 2:**
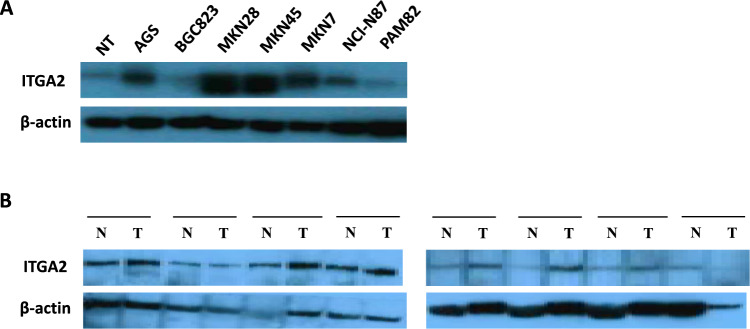
Expression of ITGA2 in gastric cancer cells and cell culture medium. Expression of ITGA2 was evaluated in gastric cancer cell lines (**A**) and tissue samples (**B**) by western blot. The results were consistent with the qPCR expression of ITGA2

Protein expression of ITGA2 was evaluated by western blot. The result was consistent with that in RT-qPCR for cell lines and tissue samples (Fig. 2A, B). It was found that protein expression of ITGA2 was high in exosomes of BCG23 culture medium. This suggested that ITGA2 could be secreted from gastric cancer cells into cell culture medium. This might be one of the reasons of low expression of ITGA2 in BCG23 cells. But its expression was not actually downregulated in this cell line, just translocated into the extracellular environment.

### Knockdown of ITGA2 inhibited gastric cancer cell proliferation

**Fig. 3:**
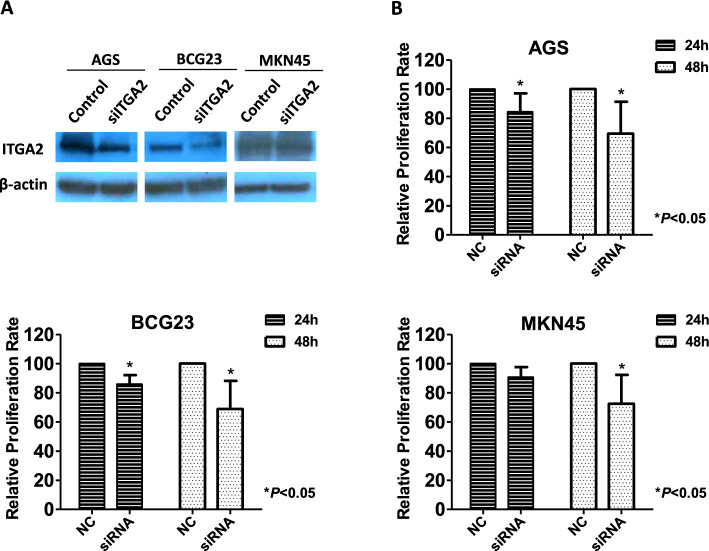
Effect of knockdown of ITGA2 in gastric cancer cell proliferation. **A** Transfection of control and siRNA of ITGA2 in AGS, BCG23, and MKN45 cells for 72 h. Downregulation of ITGA2 was observed in AGS, BCG23 and MKN45 cells. **B** Knockdown of ITGA2 significantly inhibited cell proliferation in AGS and BCG23 in 24 and 48 h, and in MKN45 in 48 h (*P < 0.05)

siRNA of ITGA2 was transfected into gastric cancer cell lines, including AGS, BCG23, and MKN45. Expression of ITGA2 in the transfected cell lines was evaluated by western blot. The result indicated that knockdown of ITGA2 in AGS, BCG23, or MKN45 was dramatically decreased and at least lasted for 72 h (Fig. 3A). The transfected cell lines were then subjected to functional assays. Cells with ITGA2 siRNA or scrambled control were subjected to MTT assay to evaluate cell proliferation. The result showed that knockdown of ITGA2 significantly inhibited gastric cancer cell proliferation by around 15–20% in 24 h and around 30% in 48 h (*P < 0.05, Fig. 3B).

### Knockdown of ITGA2 inhibited gastric cancer cell migration and invasion

**Fig. 4:**
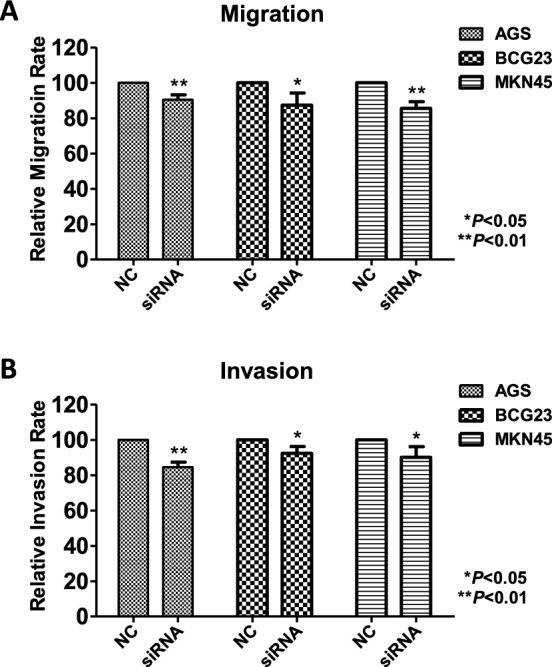
Effect of knockdown of ITGA2 in gastric cancer cell migration and invasion. Knockdown of ITGA2 significantly inhibited cell migration (**A**) and cell invasion (**B**) in AGS, BCG23, and MKN45 cells. The relative migration and the invasion rate from the fluorescence plate reader were indicated (*P < 0.05, **P < 0.01)

Cells were pre-treated with mitomycin to inhibit cell proliferation before subjecting to wound healing assay, migration, or invasion assay. Would healing assay suggested that knockdown of ITGA2 inhibited cell migration in AGS cells (Supplementary Fig. 1). The migrated cells were detached, lysed, and stained. The stained cells were red with a fluorescence plate reader at 480 nm. The migration assay indicated that migrated gastric cancer cells were significantly decreased by around 15% with knockdown of ITGA2 in AGS, BCG23, and MKN45 cells (*P < 0.05, **P < 0.01, Fig. 4A). For invasion assay, the transwell chambers were coated with Matrigel to mimic the situation of basement membrane in tumor microenvironment. The invasion assay showed that the invasive gastric cancer cells were significantly decreased with knockdown of ITGA2 by 10–20% (*P < 0.05, **P < 0.01, Fig. 4B). The above results revealed that knockdown of ITGA2 inhibited gastric cancer cell proliferation, migration, and invasion.

### Proteomic profiling for downstream targets of ITGA2 in gastric cancer

**Fig. 5:**
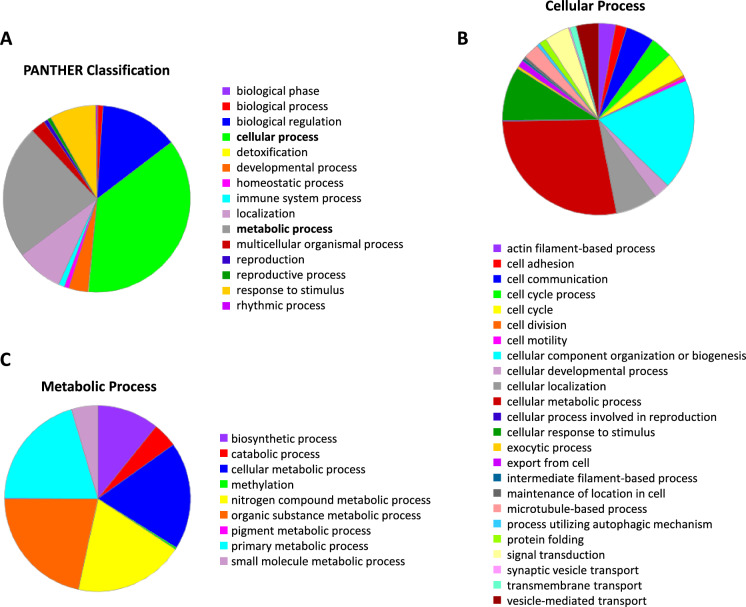
Targets of ITGA2 and relevant functions in gastric cancer. **A** Biological classification of targets releasing from proteomic profiling of knockdown of ITGA2 in gastric cancer cells. Relevant functions of cellular process and metabolic process are indicated in **B** and **C**

Proteomic profiling was performed in AGS and BCG23 cells with knockdown of ITGA2 in order to find out the downstream targets of ITGA2 in gastric cancer (Supplementary Fig. 2). The results showed that there were 1345 targets of ITGA2 in AGS cell line and 2270 targets of ITGA2 in BCG23 cell line. There were 469 targets overlapping in these two cell lines (Supplementary Table 1). These overlapping targets belong to various cell signaling and pathways, including cellular process, immune system process, metabolic process, etc. (Fig. 5A), according to PANTHER Classification System (PANTHER19.0). Classification of cellular process and metabolic process were further investigated (Fig. 5B, C).

### Targets and associated signaling pathways of ITGA2 in gastric cancer

Among the targets releasing from proteomic profiling, we found quite a number of them are critical in gastric cancer development. Clusters of them belonged to certain protein families, such as DDXs (ATP-dependent RNA helicase), KIFs (kinesin-like protein), and RMBs (RNA-binding protein). Some targets play significant roles in cell cycle and cell proliferation, including CCNB1 and its upstream factor OGFOD1. In addition, PTPN2 also attracted the attention as it exerted functions in immunotherapy. Moreover, SNRPA1 regulated a pro-metastatic splicing program. UBL3 functioned in protein sorting to small extracellular vesicles and it might affect the types and amount of the proteins packed into extracellular vesicles. The promising targets were summarized in Table 2.

**Table 2:** Promising downstream targets of ITGA2 in gastric cancer

Gene names	Protein descriptions	Gene names	Protein descriptions
ABCF2	ATP-binding cassette sub-family F member 2	LIG1	DNA ligase 1
ACSL3	Fatty acid CoA ligase Acsl3	LIG3	DNA ligase 3
APAF1	Apoptotic protease-activating factor 1	LMNB1	Lamin-B1
APOBEC3B	DNA dC- > dU-editing enzyme APOBEC-3B	MAD2L1	Mitotic spindle assembly checkpoint protein MAD2A
ARHGAP18	Rho GTPase-activating protein 18	MARCKS	Myristoylated alanine-rich C-kinase substrate
ARMT1	Damage-control phosphatase ARMT1	MRTO4	mRNA turnover protein 4 homolog
BAZ1B	Tyrosine-protein kinase BAZ1B	MT2A	Metallothionein-2
BCCIP	BRCA2 and CDKN1A-interacting protein	NAA50	N-alpha-acetyltransferase 50
BIN1	Myc box-dependent-interacting protein 1	NAT10	RNA cytidine acetyltransferase
BYSL	Bystin	NCAPD2	Condensin complex subunit 1
CCDC137	Coiled-coil domain-containing protein 137	NCAPD3	Condensin-2 complex subunit D3
CCNB1	G_2_/mitotic-specific cyclin-B1	NOLC1	Nucleolar and coiled-body phosphoprotein 1
CDCA3	Cell division cycle-associated protein 3	NOP58	Nucleolar protein 58
CDCA5	Sororin	NUP54	Nucleoporin p54
CDK2	Cyclin-dependent kinase 2	OGFOD1	Prolyl 3-hydroxylase OGFOD1
CHKA	Choline kinase alpha	PACSIN3	Protein kinase C and casein kinase substrate in neurons protein 3
CHRAC1	Chromatin accessibility complex protein 1	PAFAH2	Platelet-activating factor acetylhydrolase 2, cytoplasmic
CHTOP	Chromatin target of PRMT1 protein	PCNA	Proliferating cell nuclear antigen
CKS1B	Cyclin-dependent kinases regulatory subunit 1	PLS3	Plastin-3
CNBP	Beta-catenin-interacting protein 1	PNN	Pinin
CRABP2	Cellular retinoic acid-binding protein 2	PNO1	RNA-binding protein PNO1
CTCF	Transcriptional repressor CTCF	POLR2A	DNA-directed RNA polymerase II subunit RPB1
CTNNBIP1	Beta-catenin-interacting protein 1	PPM1G	Protein phosphatase 1G
DAB2	Disabled homolog 2	PRPF19	Pre-mRNA-processing factor 19
DCP1A	mRNA-decapping enzyme 1A	PSMA1	Proteasome subunit alpha type-1
DDI2	Protein DDI1 homolog 2	PSME3	Proteasome activator complex subunit 3
DDX10	Probable ATP-dependent RNA helicase DDX10	PTMA	Prothymosin alpha
DDX17	Probable ATP-dependent RNA helicase DDX17	PTPN2	Tyrosine-protein phosphatase non-receptor type 2
DDX20	Probable ATP-dependent RNA helicase DDX20	RBM14	RNA-binding protein 14
DDX21	Nucleolar RNA helicase 2	RBM15	RNA-binding protein 15
DDX39A	ATP-dependent RNA helicase DDX39A	RBM17	Splicing factor 45
DDX39B	Spliceosome RNA helicase DDX39B	RBM39	RNA-binding protein 39
DDX56	Probable ATP-dependent RNA helicase DDX56	RBM42	RNA-binding protein 42
DHRS1	Dehydrogenase/reductase SDR family member 1	RCC2	Protein RCC2
DIMT1	Probable dimethyladenosine transferase	RFC3	Replication factor C subunit 3
DKC1	H/ACA ribonucleoprotein complex subunit DKC1	RPA2	Replication protein A 32 kDa subunit
DLGAP5	Disks large-associated protein 5	RRM1	Ribonucleoside-diphosphate reductase large subunit
DNAJB1	DnaJ homolog subfamily B member 1	RRM2	Ribonucleoside-diphosphate reductase subunit M2
EEF1G	Elongation factor 1-gamma	SERBP1	SERPINE1 mRNA-binding protein 1
EIF3I	Eukaryotic translation initiation factor 3 subunit I	SMC2	Structural maintenance of chromosomes protein 2
EIF4ENIF1	Eukaryotic translation initiation factor 4E transporter	SNRPA1	U2 small nuclear ribonucleoprotein A
EIF5	Eukaryotic translation initiation factor 5	SRSF7	Serine/arginine-rich splicing factor 7
ESRP2	Epithelial splicing regulatory protein 2	SUZ12	Polycomb protein SUZ12
FAM111B	Serine protease FAM111B	TACC3	Transforming acidic coiled-coil-containing protein 3
FANCI	Fanconi anemia group I protein	TCOF1	Treacle protein
FEN1	Flap endonuclease 1	TIPRL	TIP41-like protein
FERMT1	Fermitin family homolog 1	TMPO	Lamina-associated polypeptide 2, isoform alpha
GORASP2	Golgi reassembly-stacking protein 2	TOP2A	DNA topoisomerase 2-alpha
GSTM3	Glutathione S-transferase Mu 3	TPD52L2	Tumor protein D54
H1-10	Histone H1.10	TPX2	Targeting protein for Xklp2
H1-2	Histone H1.2	TRMT6	tRNA (adenine(58)-N(1))-methyltransferase non-catalytic subunit TRM6
HAT1	Histone acetyltransferase type B catalytic subunit	TRMT61A	tRNA (adenine(58)-N(1))-methyltransferase catalytic subunit TRMT61A
HMGB1	High mobility group protein B1	TUBG1	Tubulin gamma-1 chain
HMGB2	High mobility group protein B2	UBE2S	Ubiquitin-conjugating enzyme E2 S
HSPA14	Heat shock 70 kDa protein 14	UBE2V1	Ubiquitin-conjugating enzyme E2 variant 1
IQGAP3	Ras GTPase-activating-like protein IQGAP3	UBL3	Ubiquitin-like protein 3
KIF11	Kinesin-like protein KIF11	USP19	Ubiquitin carboxyl-terminal hydrolase 19
KIF23	Kinesin-like protein KIF23	WDHD1	WD repeat and HMG-box DNA-binding protein 1
KIF2C	Kinesin-like protein KIF2C	WDR43	WD repeat-containing protein 43
KIFC1	Kinesin-like protein KIFC1	WDR5	WD repeat-containing protein 5
KLF5	Krueppel-like factor 5	XRCC4	DNA repair protein XRCC4
KPNA2	Importin subunit alpha-1	YBX3	Y-box-binding protein 3
KRT10	Keratin, type I cytoskeletal 10	YTHDF3	YTH domain-containing family protein 3
LASP1	LIM and SH3 domain protein 1	YY1	Transcriptional repressor protein YY1

## Discussion

In this study, we found that ITGA2 was significantly upregulated in both gastric cancer cell lines and tissue samples. Knockdown of ITGA2 suppressed gastric cancer cell proliferation, migration, and invasion, indicating that ITGA2 functions as an oncogene in gastric cancer. Notably, exosomal ITGA2 was highly expressed in the conditioned medium of gastric cancer cells, suggesting that ITGA2 can be secreted into the extracellular environment via exosomes. This secretion may contribute to the dissemination of ITGA2 from gastric cancer cells to distant sites through circulation.

Furthermore, exosomal ITGA2 levels in the BCG23 culture medium were higher than its intracellular expression, supporting the notion that ITGA2 is actively released from BCG23 cells via exosomes. These exosomes can travel to distant sites and deliver ITGA2 into recipient cells. This mechanism may help explain the elevated ITGA2 expression observed in MKN45 cells, which were derived from a liver metastatic site.

Previous studies have shown that ITGA2 acts as an oncogene in various cancers, including breast cancer [19], liver cancer [21], lung cancer [22], and colorectal cancer [27, 28]. Additionally, downstream targets and associated signaling pathways of ITGA2 have been reported to regulate numerous cellular functions in cancers. For instance, one study found that high ITGA2 expression modulates the levels of MET, PD-L1, CD4, and CD8 within the immune microenvironment in pancreatic cancer [29]. Another study demonstrated that ITGA2 overexpression promotes tumor aggressiveness by upregulating PD-L1 expression through activation of the STAT3 signaling pathway [25].

Through proteomic profiling, this study identified a panel of high-confidence downstream targets of ITGA2. These targets are functionally enriched in cell cycle regulation, cell adhesion and motility, cancer initiation and progression, and exosome-mediated vesicle transport. Notably, several belong to protein families with well-established oncogenic roles in gastric cancer. For example, DDX family members DDX21 and DDX56 have been shown to promote gastric cancer progression [30, 31]. CCNB1 is a key regulator of gastric cancer cell growth, proliferation, and metastasis [32, 33], and OGFOD1 functions upstream of CCNB1 to control cell proliferation [34, 35]. These findings support the existence of a functionally important ITGA2–OGFOD1–CCNB1 axis in gastric carcinogenesis. In addition, proteins, such as RCC2, TOP2A, WDR5, and YTHDF3, have been strongly linked to progression in gastric cancer [36–40].

This study demonstrates that exosomes can regulate the endogenous expression of ITGA2 in gastric cancer cells. We confirmed that ITGA2 is significantly upregulated in gastric cancer and functions as an oncogene by promoting tumor growth, invasion, and metastasis through multiple signaling pathways. To our knowledge, this is the first study to systematically investigate both the functional roles of ITGA2 and its regulation by tumor-derived exosomes in gastric cancer. These findings deepen our understanding of the molecular mechanisms underlying gastric carcinogenesis and identify the exosomes–ITGA2 axis as a promising therapeutic target for gastric cancer.

## Supplementary Information

Below is the link to the electronic supplementary material.

Supplementary file1 (DOCX 19 kb)

Supplementary file2 (PDF 218 kb)

## Data Availability

All data generated or analyzed during this study are included in this article and its supplementary material files. Further enquiries can be directed to the corresponding author.
