# Supplementary material for: Proteomic profiling identifies an oncogene ITGA2 and its downstream targets in gastric cancer
Source: Clin Transl Oncol. 2026 Feb 2;28(7):2792–802. doi: 10.1007/s12094-026-04231-w (PMC13282363; doi:10.1007/s12094-026-04231-w)
Supplement: Supplementary file 1 — Supplementary file1 (DOCX 19 kb) [file 12094_2026_4231_MOESM1_ESM.docx]

Supplementary Table 1. Proteins overlapping in AGS and BCG23 cells with knockdown of ITGA2.

| ABCF2 | CALD1 | DCP1A | ENO3 | GLS | KIFC1 | MEPCE | NPLOC4 | PNO1 | RPA2 | SLC43A2 | TMEM87A |
| --- | --- | --- | --- | --- | --- | --- | --- | --- | --- | --- | --- |
| ABCF3 | CAMK2D | DDAH1 | EPHX1 | GNAI2 | KLF5 | MICALL1 | NT5C | POLR1C | RPL17 | SLC44A2 | TMEM87B |
| ACAT2 | CAP2 | DDB1 | ERAL1 | GNS | KPNA2 | MINK1 | NUDC | POLR2A | RPL18 | SLC4A1AP | TMPO |
| ACOX1 | CAT | DDB2 | ERAP1 | GOLM1 | KRT10 | MINPP1 | NUDT15 | POLR2I | RPL24 | SMC2 | TOP2A |
| ACSL3 | CCDC137 | DDI2 | ERLIN2 | GORASP2 | KRT80 | MMP14 | NUP35 | PPCS | RPL28 | SMS | TPD52L2 |
| ACTBL2 | CCDC22 | DDRGK1 | ESRP2 | GPKOW | L1CAM | MORF4L1 | NUP54 | PPIE | RPL29 | SMU1 | TPP1 |
| ADAM10 | CCDC47 | DDX10 | ETFB | GPRC5A | LAMB1 | MRPL39 | OAS1 | PPM1G | RPL3 | SNAP23 | TPRG1L |
| ADAM9 | CCDC51 | DDX17 | EXOC4 | GRN | LAMC1 | MRPS14 | OASL | PPM1H | RPL32 | SNRNP200 | TPX2 |
| AHCYL1 | CCNB1 | DDX20 | EXOSC2 | GSS | LAMP1 | MRPS36 | OGA | PPP1CC | RPL34 | SNRPA1 | TRAPPC2L |
| AHNAK | CD276 | DDX21 | FAM111B | GSTM3 | LAMTOR1 | MRTFA | OGFOD1 | PPP1R12C | RPL36 | SNTB2 | TRIR |
| AHNAK2 | CD44 | DDX39A | FAM234A | GUK1 | LASP1 | MRTO4 | P4HA2 | PPP1R21 | RPL4 | SNX17 | TRMT6 |
| AKT1 | CD55 | DDX39B | FAM98B | H1-10 | LGALS3BP | MT2A | PACSIN3 | PPWD1 | RPL8 | SOAT1 | TRMT61A |
| ALDH3A1 | CD59 | DDX56 | FANCI | H1-2 | LIG1 | MTHFR | PAFAH2 | PRKAR1A | RPL9 | SPNS1 | TSC2 |
| ALDH9A1 | CDC42EP1 | DECR1 | FAR1 | H1-4 | LIG3 | MTPN | PARP1 | PRPF19 | RPP30 | SPPL2B | TSPAN13 |
| ANKMY2 | CDCA3 | DEK | FBL | HADHA | LIMA1 | MTX2 | PARP4 | PRPF3 | RPS25 | SQSTM1 | TSR1 |
| APAF1 | CDCA5 | DERA | FBLN1 | HAT1 | LIMS1 | MVP | PAXX | PRPSAP1 | RPS3A | SRC | TTN |
| APLP2 | CDK2 | DHRS1 | FBXO30 | HERC4 | LMNB1 | MYH9 | PBXIP1 | PSMA1 | RPS8 | SRGAP2 | TUBB2A |
| APOBEC3B | CEMIP2 | DHX36 | FDFT1 | HINT1 | LSR | MYL9 | RAB11FIP1 | PSMA4 | RRM1 | SRI | TUBG1 |
| APP | CERS2 | DHX38 | FDXR | HMGB1 | LUC7L3 | NAA50 | RAB27B | PSME3 | RRM2 | SRSF7 | TXNDC12 |
| ARHGAP18 | CETN2 | DIMT1 | FEN1 | HMGB2 | LYPLAL1 | NACA | RAB3D | PSME3IP1 | RSL24D1 | STX12 | TXNDC9 |
| ARMT1 | CHKA | DKC1 | FERMT1 | HMGCL | M6PR | NACA4P | RALGAPB | PSMF1 | RTN4 | STX18 | TXNRD1 |
| ARPC1A | CHRAC1 | DLGAP5 | FGG | HMOX1 | MAD2L1 | NADK2 | RAVER1 | PSTPIP2 | TACC3 | STX7 | TYMS |
| ARPC5L | CHTOP | DNAJB1 | FHIP2A | HOOK3 | MAGOHB | NAGK | RBM14 | PTK7 | TACSTD2 | STXBP1 | UBE2C |
| ATL3 | CHURC1 | DNAJC13 | FHL1 | HSPA14 | MAN2A1 | NaN | RBM15 | PTMA | TAGLN | SUCLG2 | UBE2S |
| ATP6V1E1 | CKS1B | DNAJC3 | FKBP8 | ID1 | MAP4K5 | NAPA | PCNA | PTPN2 | TAPBP | SURF6 | UBE2T |
| ATXN10 | CNBP | DNAJC5 | FLOT1 | IDH2 | MAPKAPK2 | NAT10 | PCYOX1 | S100A13 | TAX1BP3 | SUZ12 | UBE2V1 |
| BAG3 | CORO2A | DNTTIP2 | FLOT2 | IDI1 | MAPRE2 | NAT14 | PDS5A | SCAF1 | TCOF1 | SYNGR2 | UBL3 |
| BAIAP2 | CPTP | DUT | FNBP1L | IFIT3 | MARCKS | NCAPD2 | PEA15 | SCD | TFDP1 | VASN | UBXN4 |
| BAZ1B | CRABP2 | DYNC1LI1 | FNDC3B | IFITM3 | MBOAT7 | NCAPD3 | PEBP1 | SCP2 | SEZ6L2 | VAT1 | UFL1 |
| BCAP31 | CSDE1 | DYNLL2 | FSTL1 | IK | MCAT | NDUFA8 | PFKL | SDF4 | SF3A2 | VCL | UMPS |
| BCAT2 | CTCF | EEF1G | FTH1 | IL18 | MCCC1 | NDUFB11 | PFKP | SEC23IP | SFN | WARS2 | USP19 |
| BCCIP | CTNNBIP1 | EEFSEC | FTL | INCENP | MCM2 | NDUFS5 | PGM2 | SERBP1 | SFT2D2 | WDHD1 | USP47 |
| BIN1 | CTPS2 | EIF2AK2 | FXR1 | IQGAP3 | MCM3 | NHP2 | PI4KB | RBM17 | SLAIN2 | WDR43 | UTP3 |
| BMP1 | CTSD | EIF3F | GALE | ITGA5 | MCM4 | NIPSNAP2 | PICALM | RBM39 | SLC16A3 | WDR5 | WIPI2 |
| BST2 | CTSZ | EIF3G | GAR1 | ITGB1 | MCM5 | NME3 | PITPNA | RBM42 | SLC30A7 | THNSL1 | XRCC4 |
| BUD31 | CXADR | EIF3I | GC | JUN | MCM6 | NOLC1 | PLD3 | RCC1 | SLC35B2 | THOC3 | YARS1 |
| BYSL | DAB2 | EIF4ENIF1 | GHITM | KIF11 | MCM7 | NOP56 | PLPP2 | RCC2 | SLC35F6 | THOC6 | YBX3 |
| C7 | DAP3 | EIF5 | GINS1 | KIF23 | MCRIP2 | NOP58 | PLS3 | RFC3 | SLC38A2 | TIPRL | YTHDF2 |
| C9 | DBT | EML2 | GIPC1 | KIF2C | MEA1 | NPC2 | PNN | ROCK1 | SLC39A1 | TMEM41B | YTHDF3 |
| YY1 | ZNF217 | ZNF598 |  |  |  |  |  |  |  |  |  |
