# Supplementary material for: Proteomic profiling identifies an oncogene ITGA2 and its downstream targets in gastric cancer
Source: Clin Transl Oncol. 2026 Feb 2;28(7):2792–802. doi: 10.1007/s12094-026-04231-w (PMC13282363; doi:10.1007/s12094-026-04231-w)
Supplement: Supplementary file 2 — Supplementary file2 (PDF 218 kb) [file 12094_2026_4231_MOESM2_ESM.pdf]

Supplementary Figure 1.

Wound healing assay of gastric cancer cells with knockdown of ITGA2 in AGS cells

AGS

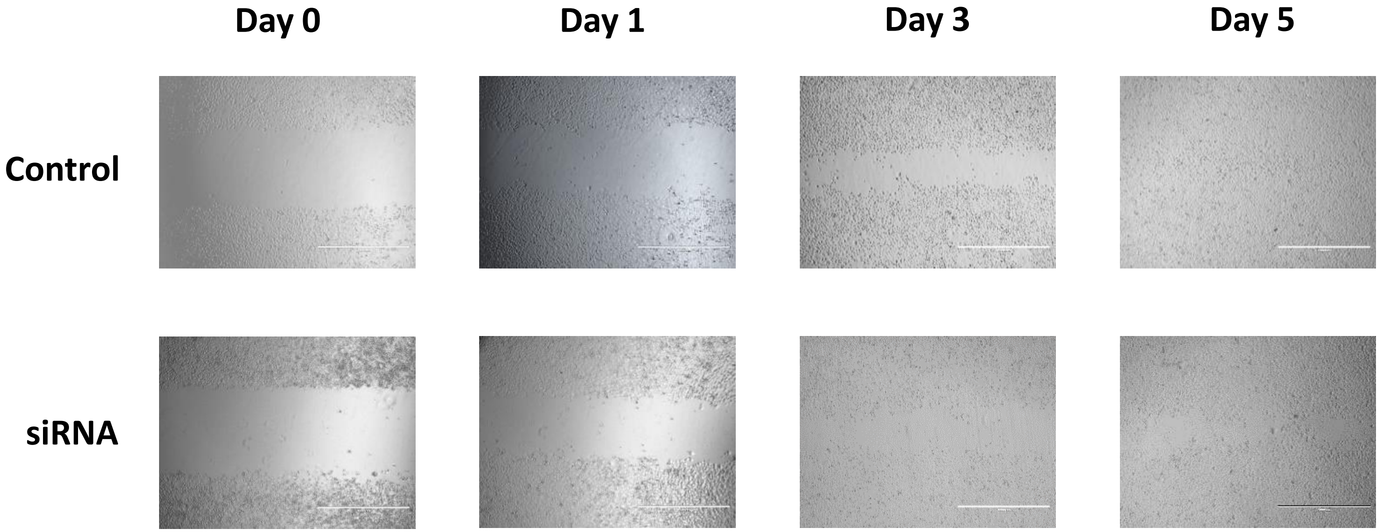

Supplementary Figure 2.

Proteomics profiling in AGS and BCG23 cells with knockdown of ITGA2

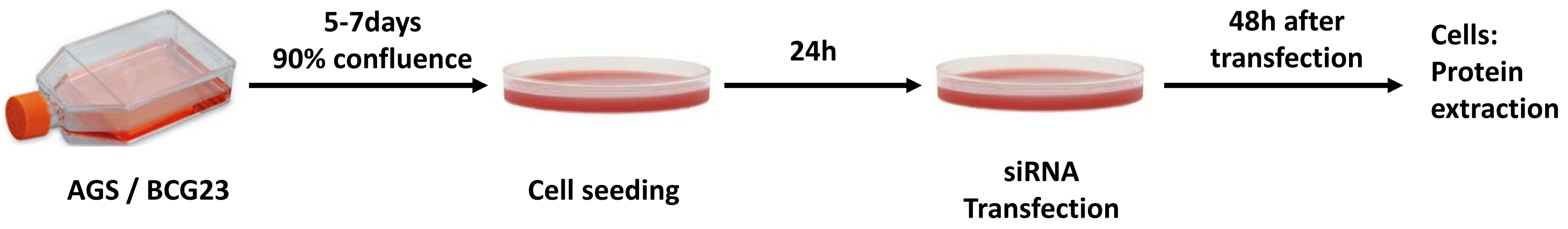

Proteins
1 million cells/sample, 2 samples/cell line, triplicated

⇩

Label Free Quantitative Proteomics

⇩

Liquid chromatography-mass spectrometry
LC-MS/MS using Bruker timsTOF pro mass spectrometry
interfaced with nanoelute UHPLC
Data Analysis - LFQ-DIA
